# Correction to ‘Ubiquitinated histone H2B as gatekeeper of the nucleosome acidic patch’

**DOI:** 10.1093/nar/gkae751

**Published:** 2024-09-01

This is a correction to: Chad W Hicks, Sanim Rahman, Susan L Gloor, James K Fields, Natalia Ledo Husby, Anup Vaidya, Keith E Maier, Michael Morgan, Christopher-Michael Keogh, Cynthia Wolberger, Ubiquitinated histone H2B as gatekeeper of the nucleosome acidic patch, *Nucleic Acids Research*, 2024; https://doi.org/10.1093/nar/gkae698

In the originally published version of the manuscript, the ninth author was omitted from the author list. Their name and affiliation have now been added to the authorlist.

In the **References** list, errors in page numbers, authors’ initials, citations and links were emended as this would affect readers’ searches and further citation.

These emendations have been made in the article.

