# Supplementary material for: Ubiquitinated histone H2B as gatekeeper of the nucleosome acidic patch
Source: Nucleic Acids Res. 2024 Aug 16;52(16):9978–95. doi: 10.1093/nar/gkae698 (PMC11381367; doi:10.1093/nar/gkae698)
Supplement: gkae698_Supplemental_File [file gkae698_supplemental_file.pdf]

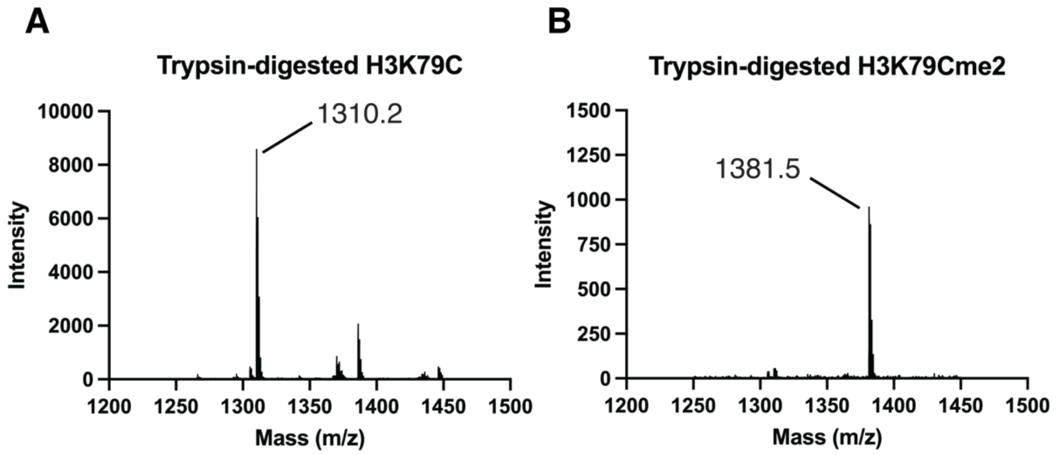

Figure S1. Mass spectrometry validation of H3K$_C$79me2. Matrix Assisted Laser Desorption/Ionization - Mass Spectrometry (MALDI-MS) analysis of trypsin-digested **(A)** unreacted H3K79C and **(B)** purified H3K$_C$79me2. Trypsin-digestion of H3K79C should produce a theoretical 1310 Da fragment while trypsin-digestion of H3K$_C$79me2 should produce a theoretical 1382 Da fragment.

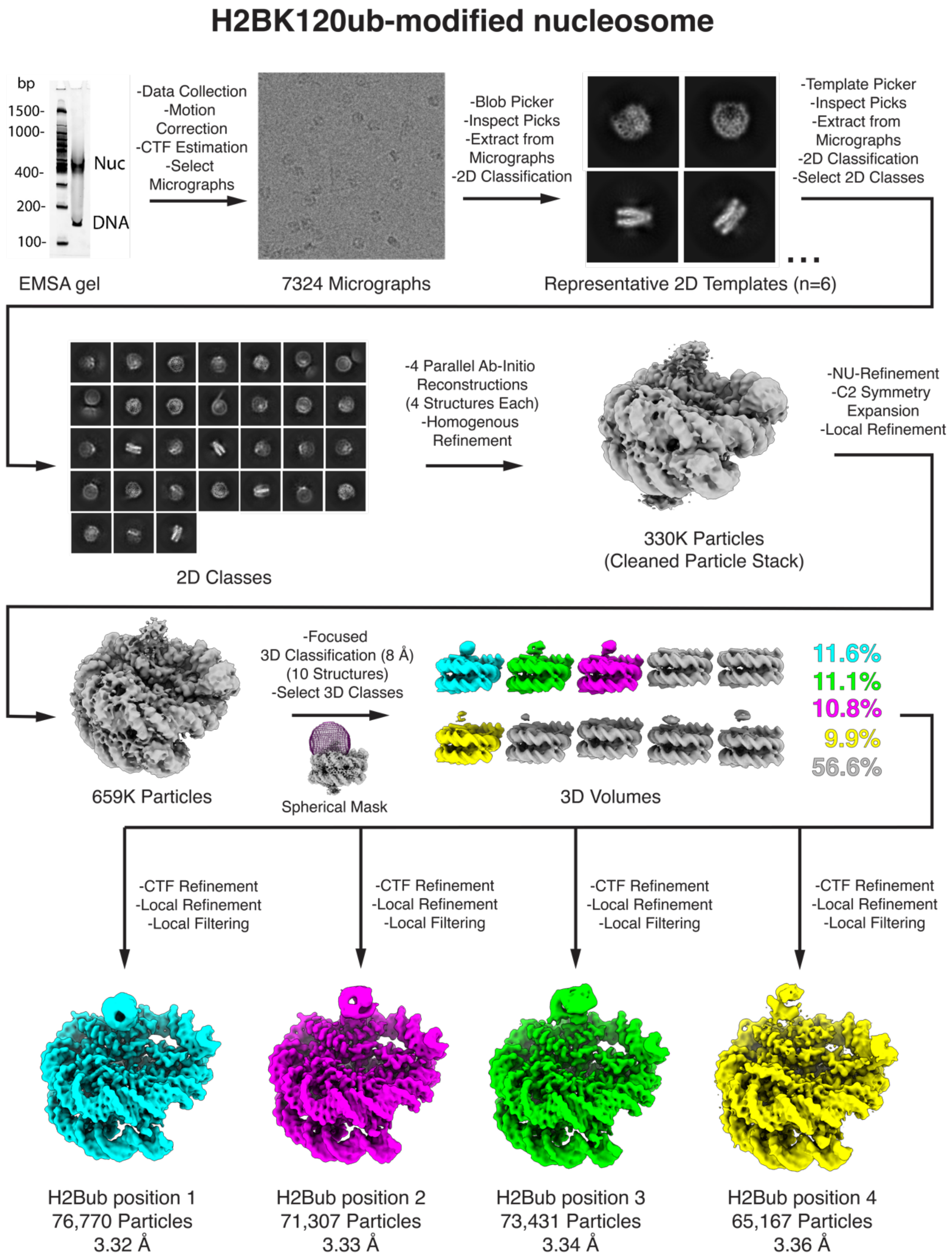

Figure S2. CryoEM data processing workflow for nucleosome containing H2BK120ub and Widom 601 DNA (147 bp).

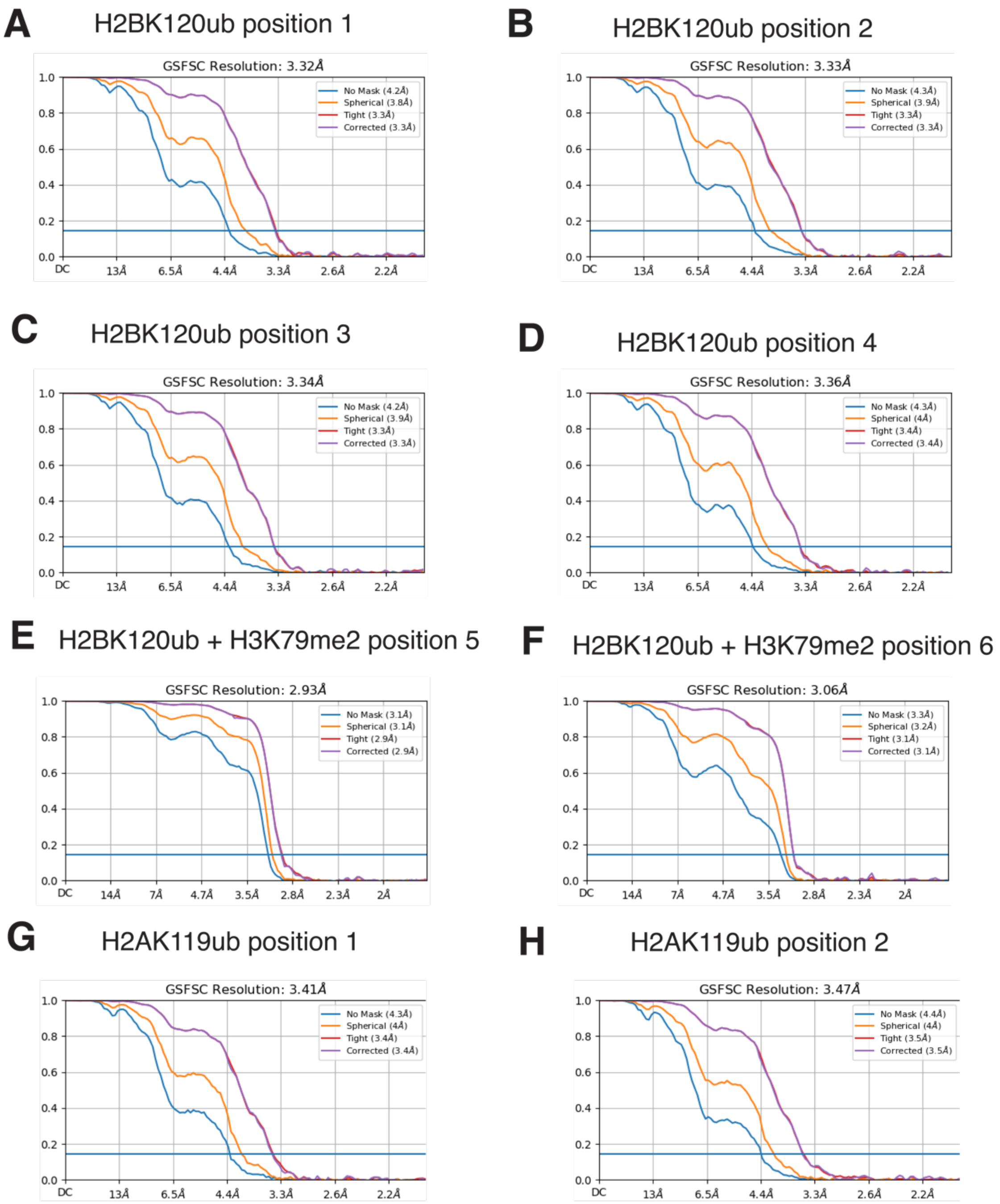

Figure S3. Fourier Shell Correlation (FSC) plots of all cryoEM maps. FSC plots using gold-standard 0.143 cutoff of cryoEM structures for: H2BK120ub nucleosome **(A-D)**, H2BK120ub+H3K_C79me2 nucleosome **(E-F)**, and H2AK119ub nucleosome **(G-H)**.

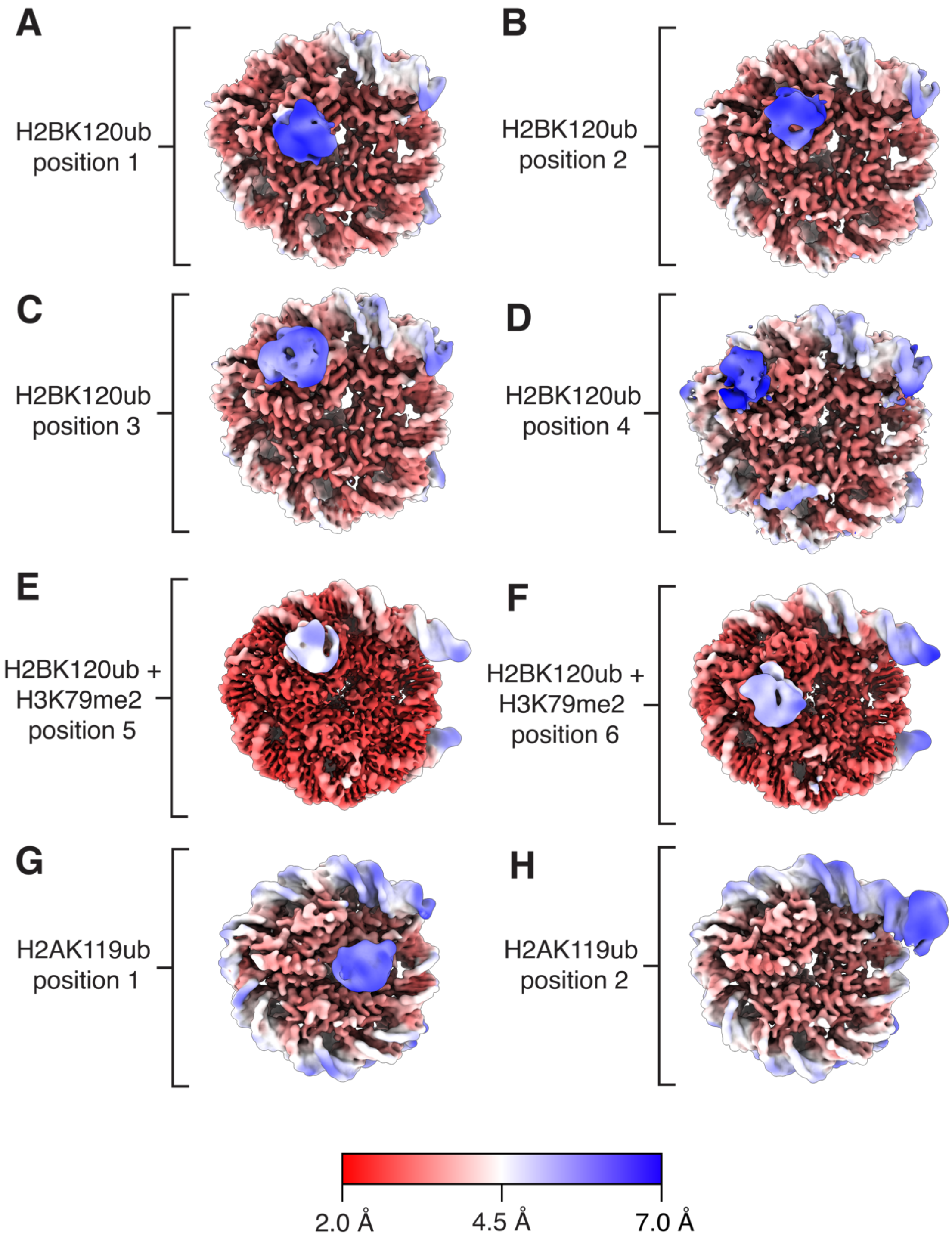

Figure S4. Local resolution estimation representations of all cryoEM maps. Local resolution estimation color depictions of cryoEM maps of H2BK120ub nucleosome **(A-D)**, H2BK120ub+H3K$_C$79me2 nucleosome **(E-F)**, and H2AK119ub nucleosome **(G-H)**.

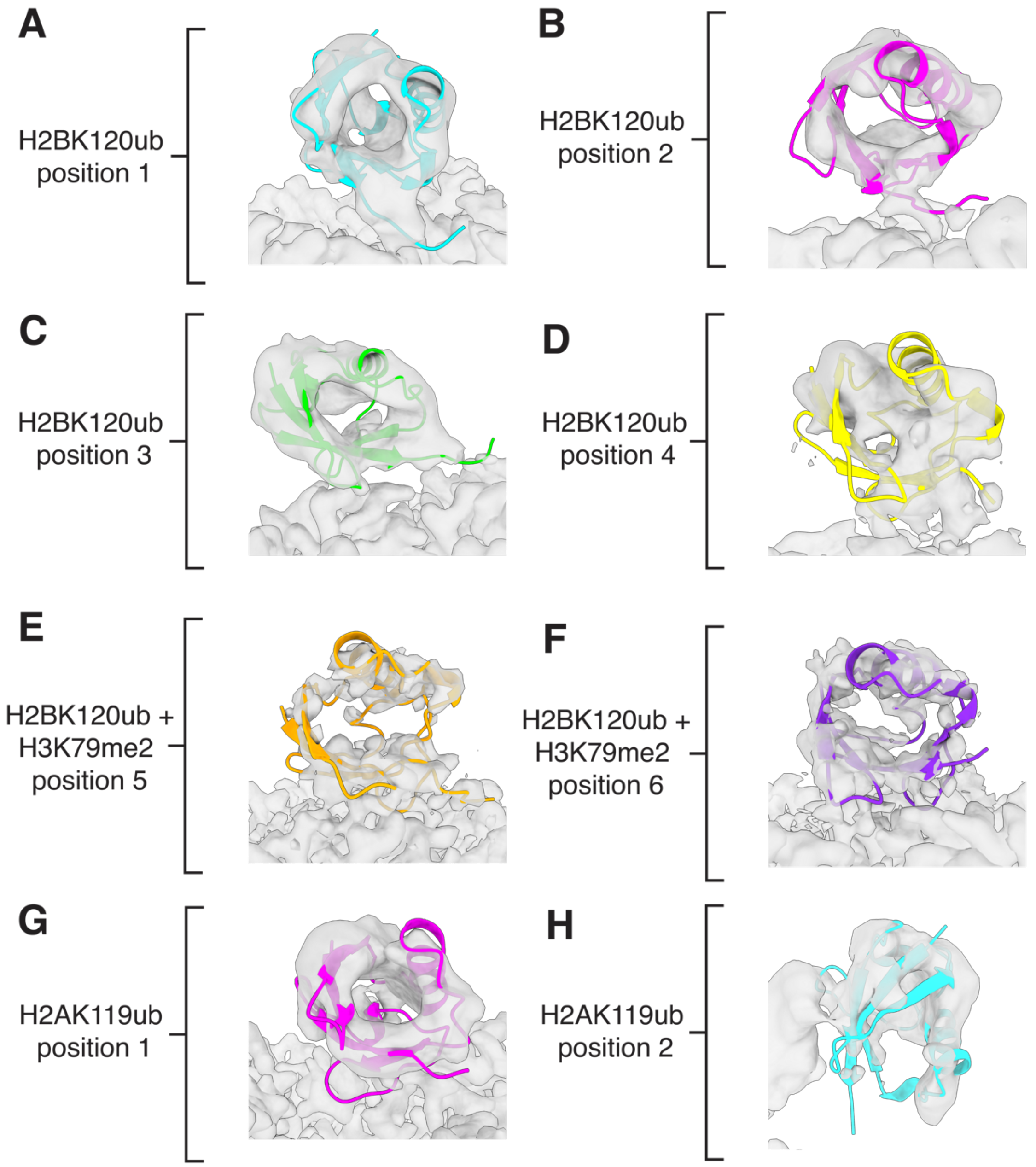

Figure S5. Fit of model to map for all structures. Ubiquitin cartoon model showing the fit to cryoEM maps corresponding to: H2BK120ub nucleosome, with ubiquitin in positions 1, 2, 3, and 4 **(A-D)**, H2BK120ub+H3K$_{C}$79me2 nucleosome with ubiquitin in positions 5 and 6 **(E-F)**, and H2AK119ub nucleosome with ubiquitin in positions 1 and 2 **(G-H)**.

A

Acidic Patch-Ubiquitin Buried Surface Area

Probe Radius (Å)	(Default) 1.4	3	4	5	6	7	8	9	10	15	20
H2Bub-Pos1 ($Å^2$)	0	0	869	1235	1583	1943	2322	2723	3146	5618	8700
H2Bub-Pos2 ($Å^2$)	0	0	422	891	1203	1591	1980	2379	2802	5230	8249
H2Bub-Pos3 ($Å^2$)	0	0	0	405	592	1176	1525	1898	2288	4642	7573
H2Bub-Pos4 ($Å^2$)	0	0	0	0	315	774	1043	1335	1664	3743	6467
H2Bub-Pos5* ($Å^2$)	0	0	390	890	1239	1599	1970	2358	2767	5175	8186
H2Bub-Pos6* ($Å^2$)	0	334	797	1119	1459	1849	2247	2657	3084	5555	8637
H2Aub-Pos1 ($Å^2$)	0	0	344	480	979	1241	1530	1844	2188	4350	7139
H2Aub-Pos2 ($Å^2$)	0	0	0	0	0	0	0	0	0	0	377

B

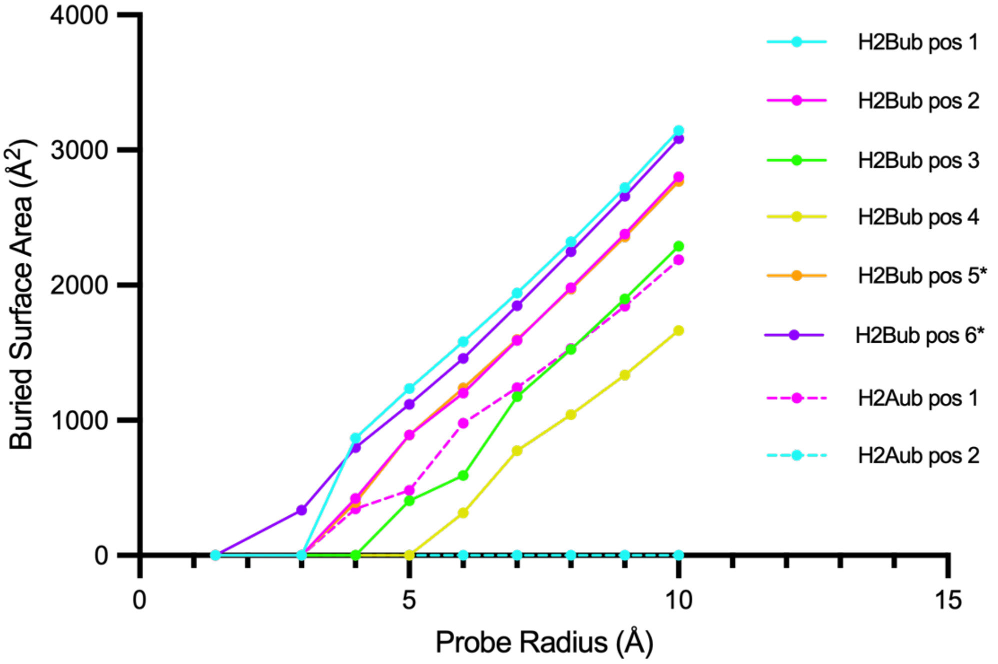

Figure S6. Acidic patch – ubiquitin buried surface area calculations for all ubiquitin positions. Full table of values **(A)** and line chart (up to 10 Å probe radius) **(B)** showing buried surface area between ubiquitin (all Ub residues) and the nucleosome acidic patch (H2A residues E56, E61, E64, D90, E91, E 92 and H2B residues E105, E113) at all ubiquitin positions. Calculated using “interfaces” command in ChimeraX at a range of probe radii. Solid lines correspond to H2Bub positions while dashed lines and square data points correspond to H2Aub positions. * indicates the H2Bub position was derived from the H2BK120ub+H3K$_C$79me2 nucleosome cryoEM dataset.

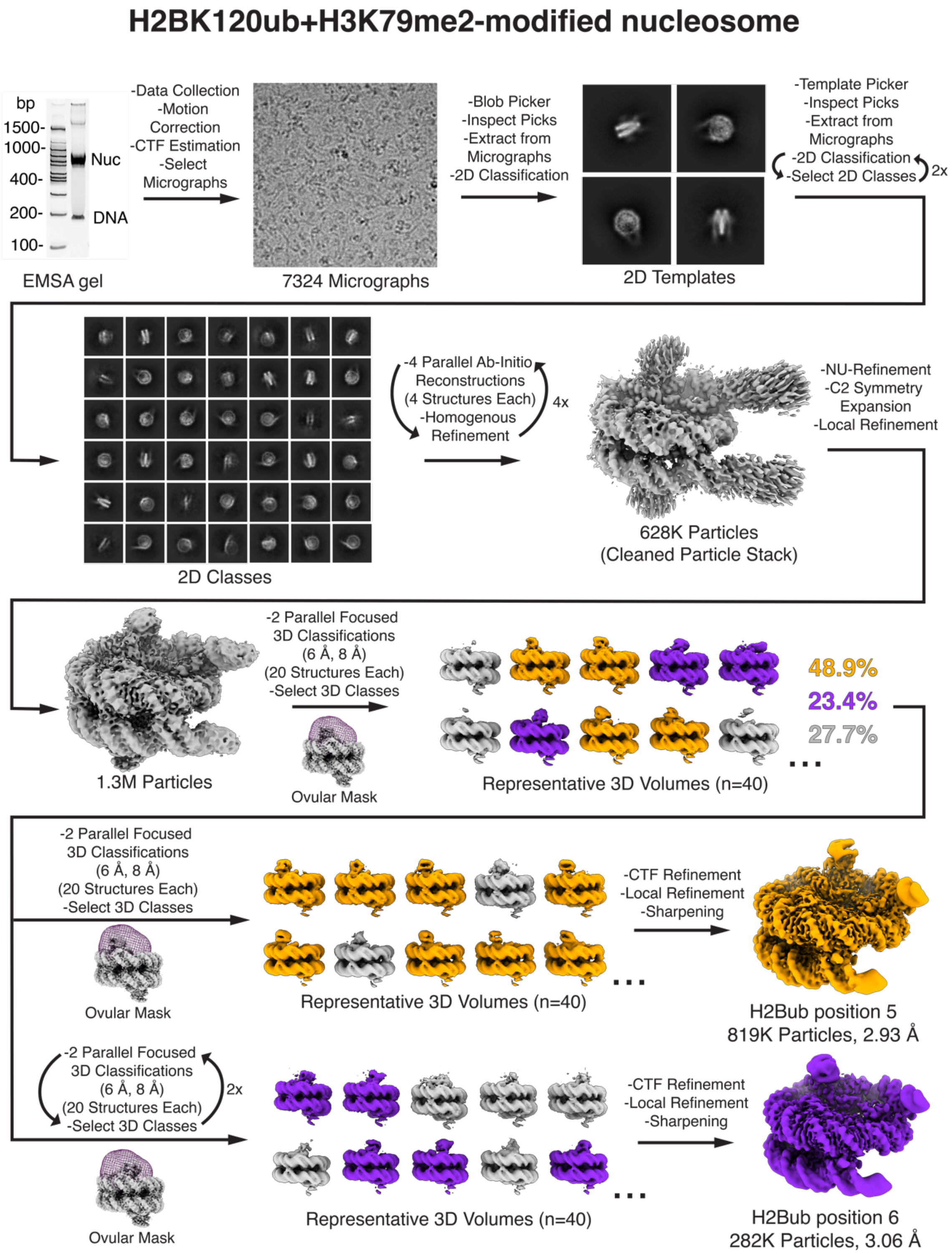

Figure S7. CryoEM data processing workflow for nucleosome containing H2BK120ub, H3K$_C$79me2, and Widom 601 DNA with 19 bp linkers (185 bp).

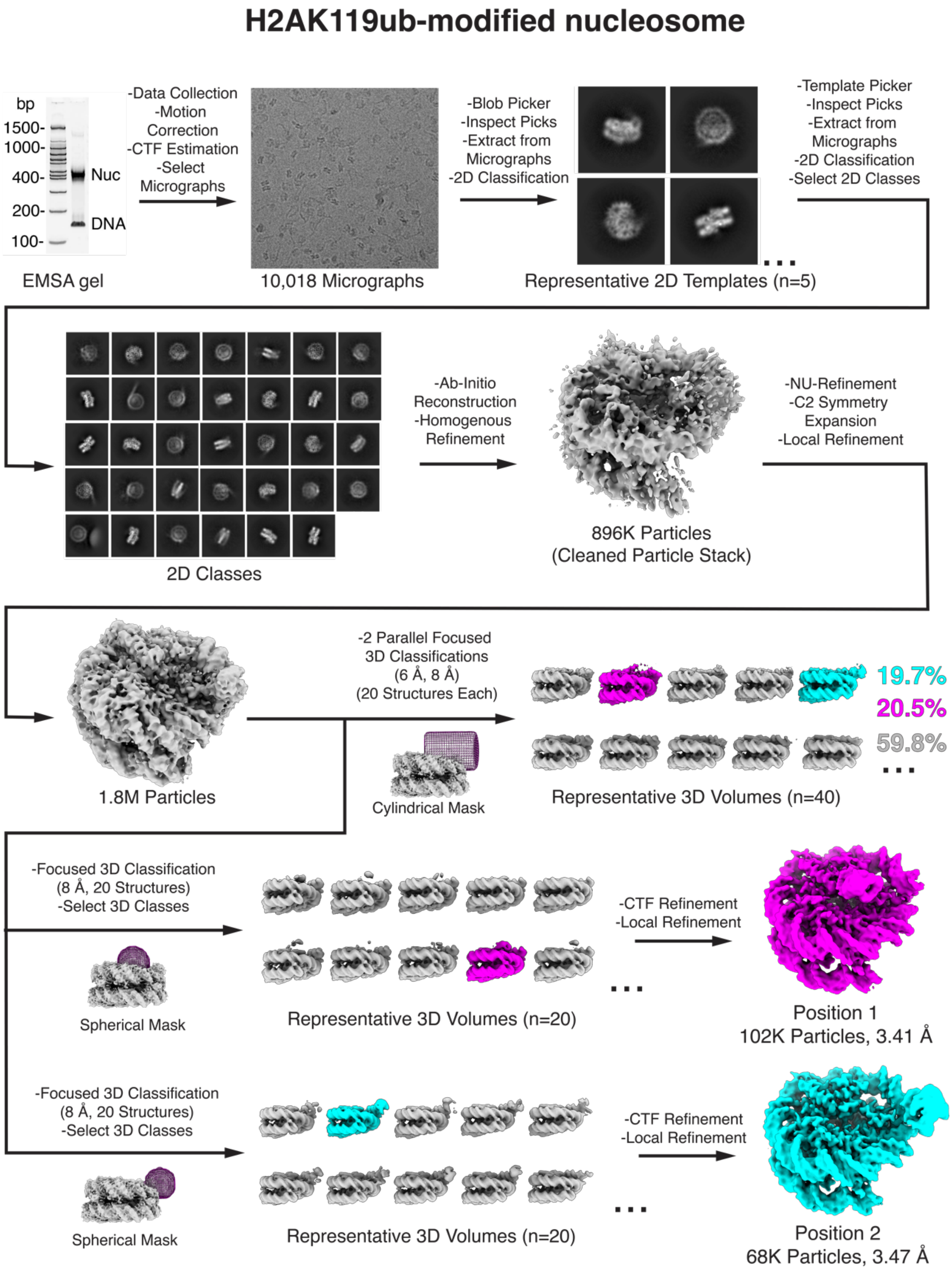

Figure S8. CryoEM data processing workflow for nucleosome containing H2AK119ub and Widom 601 DNA (147 bp).

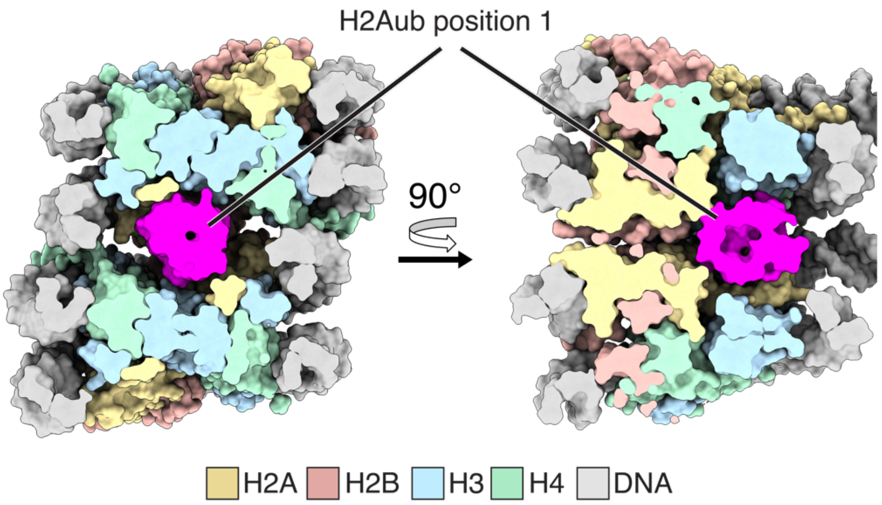

Figure S9. H2AK119ub nucleosome with ubiquitin in position 1 can accommodate nucleosome stacking without steric clash. CryoEM model of the ubiquitin of H2Aub nucleosome in position 1 (ubiquitin + lower nucleosome), depicted in surface representation, superimposed over an X-ray structure of a tetranucleosome (PDB:1ZBB). Figure is shown in a cut-away surface representation, with the slice halfway through the ubiquitin.

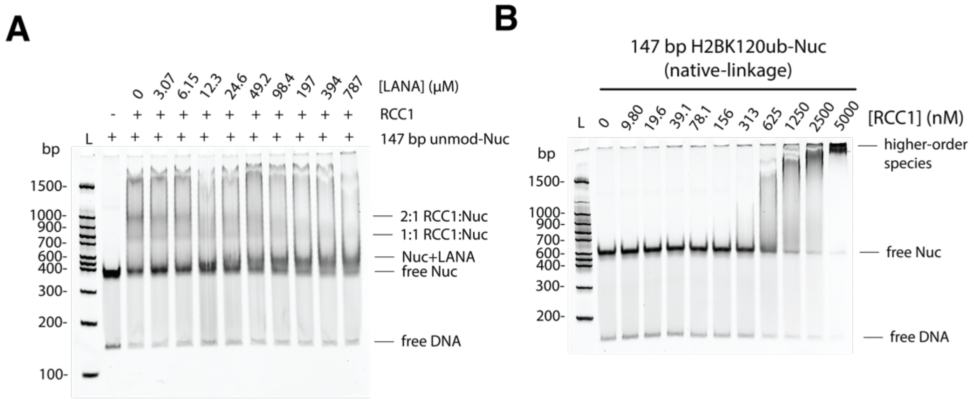

Figure S10. Binding of RCC1 to nucleosomes in the presence and absence of LANA peptide. (A) EMSA showing RCC1 (625 nM) binding to unmodified nucleosome (100 nM) in the presence of increasing amount of LANA peptide (0-787 mM). **(B)** RCC1 binding to nucleosomes containing a native linked H2BK120ub. The nucleosomes in all panels contained 147 bp Widom 601 DNA. All gels stained with SYBR Gold.

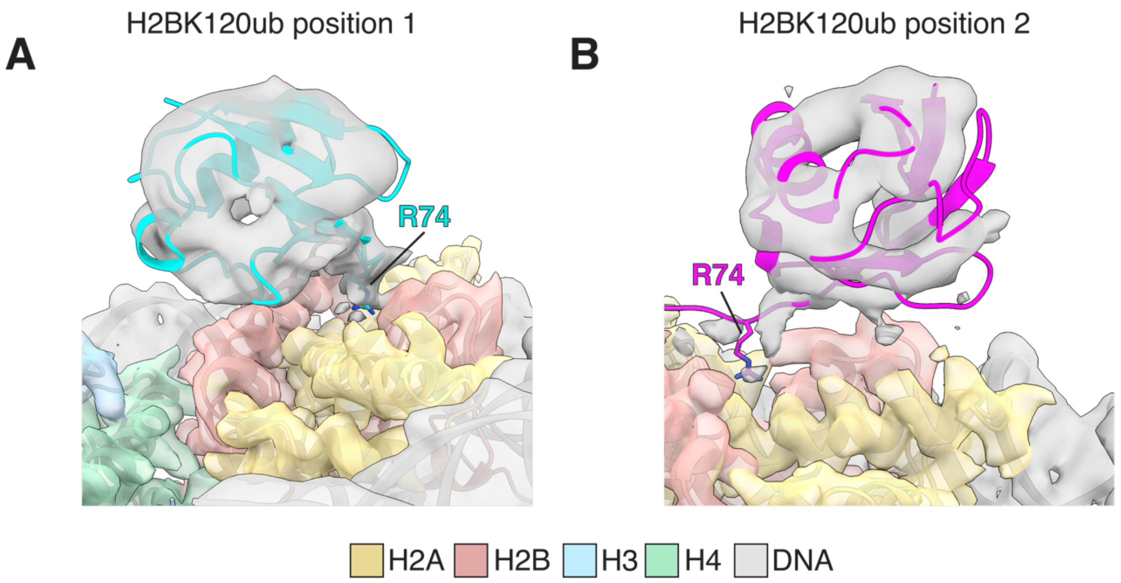

Figure S11. Arginine 74 of ubiquitin in H2BK120ub nucleosome interacts with the acidic patch formed by histone H2A/H2B. (A) R74 of ubiquitin in position 1 interacts with the nucleosome surface. **(B)** R74 of ubiquitin in position 2 interacts with the nucleosome surface.

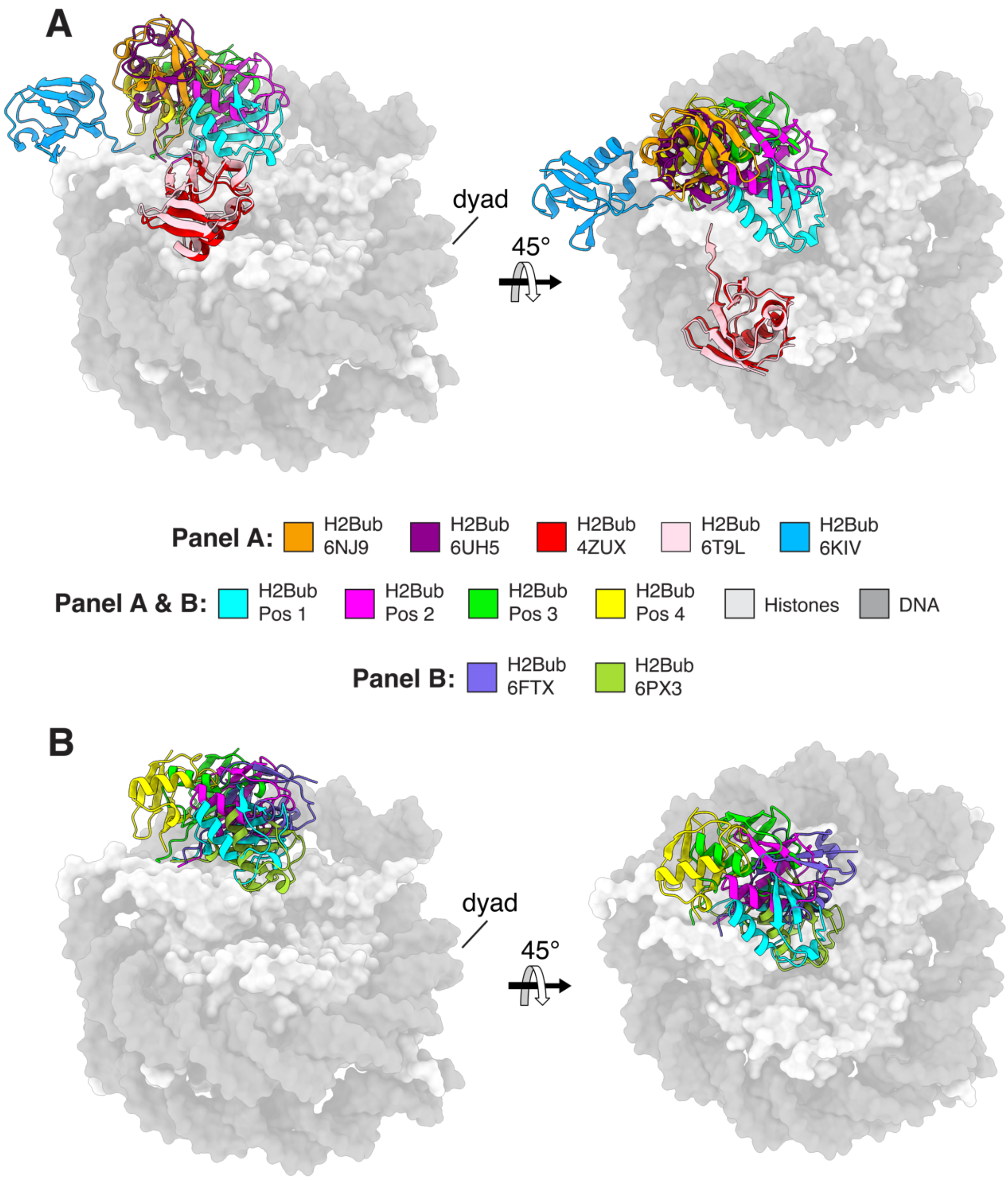

Figure S12. Ubiquitin binding proteins can greatly alter the position of H2BK120ub on the surface of the nucleosome. (A) Composite figure showing the positions of H2Bub from previously published structures. Compared to structures of H2Bub nucleosomes only (cyan, magenta, lime, yellow) (this study), H2Bub can adopt altered positions when bound by Dot1L (orange) (PDB: 6NJ9), COMPASS complex (purple) (PDB: 6UH5), SAGA DUB module (red, pink) (PDBs: 4ZUX, 6T9L), or MLL complex (sky blue) (PDB: 6KIV). **(B)** Previously published structures of H2BK120ub nucleosomes where Chd1 (PDB: 6FTX) or Set2 (PDB: 6PX3) are bound to nucleosome but not to H2BK120ub (slate blue, yellow green, respectively).

Table S1. CryoEM data collection, processing, refinement, and validation statistics

	H2BK120ub ubiquitin position 1	H2BK120ub ubiquitin position 2	H2BK120ub ubiquitin position 3	H2BK120ub ubiquitin position 4	H2BK120ub + H3K79me2 ubiquitin position 5	H2BK120ub + H3K79me2 ubiquitin position 6	H2AK119ub ubiquitin position 1	H2AK119ub ubiquitin position 2
	**Data Collection and Processing**							
**Magnification (X)**	130,000	130,000	130,000	130,000	105,000	105,000	130,000	130,000
**Voltage (kV)**	300	300	300	300	300	300	300	300
**Electron exposure ($e^-/Å^2$)**	40	40	40	40	50	50	40	40
**Dose rate (e^-/px/s)**	5.82	5.82	5.82	5.82	12.33	12.33	6.42 to 6.46	6.42 to 6.46
**Defocus range (μM)**	-0.5 to -2.5	-0.5 to -2.5	-0.5 to -2.5	-0.5 to -2.5	-0.75 to -1.75	-0.75 to -1.75	-0.2 to -5.0	-0.2 to -5.0
**Pixel size (Å)**	0.97	0.97	0.97	0.97	0.436	0.436	0.970	0.970
**Camera**	Falcon 4	Falcon 4	Falcon 4	Falcon 4	Gatan K3	Gatan K3	Falcon 4	Falcon 4
**Energy Filter slit width (eV)**	10	10	10	10	20	20	10	10
**Micrographs (#)**	8,392	8,392	8,392	8,392	7324	7324	10,018	10,018
**Initial cleaned particle stack (#)**	329,531	329,531	329,531	329,531	628,403	628,403	895,614	895,614
**Particle duplication symmetry**	C2	C2	C2	C2	C2	C2	C2	C2
**Final particles (#)**	76,770	71,307	73,431	65,167	818,874	282,239	102,259	68,407
**Map resolution at 0.143 FSC cutoff (Å)**	3.32	3.33	3.34	3.36	2.93	3.06	3.41	3.47
	**Refinement**							
**Initial models used (PDB)**	Nuc: 4ZUX Ub: 1UBQ	Nuc: 4ZUX Ub: 1UBQ	Nuc: 4ZUX Ub: 1UBQ	Nuc: 4ZUX Ub: 1UBQ	Nuc: 4ZUX Ub: 1UBQ	Nuc: 4ZUX Ub: 1UBQ	Nuc: 4ZUX Ub: 1UBQ	Nuc: 4ZUX Ub: 1UBQ
**Map resolution range (Å)**	2.91 to 28.13	2.85 to 40.28	2.88 to 40.12	2.95 to 29.43	2.39 to 8.00	2.53 to 9.18	2.94 to 30.78	2.08 to 11.05
**Model Resolution at 0.5 FSC cutoff (Å)**	3.3	3.4	3.4	3.4	3.1	3.2	3.8	3.7
**Non-hydrogen atoms (#)**	12,689	12,689	12,689	12,659	12,985	12,974	12,485	12,480
**Nucleotides (#)**	290	290	290	290	314	314	290	290
**Ligands (#)**	0	0	0	0	0	0	0	0
**Protein B factor ($Å^2$)**	80.70	85.63	81.28	120.78	8.19	34.06	139.15	129.64
**Nucleotide B factor ($Å^2$)**	118.40	123.02	121.77	126.57	27.85	79.25	188.18	184.4
**Ligand B factor ($Å^2$)**	---	---	---	---	---	---	---	---
**Bond length RMSD (Å)**	0.005	0.005	0.004	0.005	0.003	0.003	0.004	0.005
**Bond angles RMSD (°)**	0.793	0.794	0.781	0.778	0.566	0.562	0.810	0.798
	**Validation**							
**MolProbity score**	1.54	1.40	1.51	1.41	1.29	1.29	1.74	1.58
**Clashscore**	6.54	7.28	7.80	5.26	4.36	5.35	10.47	9.02
**Rotamer Outliers (%)**	0.42	0.28	0.42	0.56	1.16	0.00	0.14	0.43
**Ramachandran Favored (%)**	96.99	98.07	97.59	97.34	97.88	98.50	96.78	97.52
**Ramachandran Allowed (%)**	3.01	1.93	2.41	2.66	2.12	1.50	3.22	2.48
**Ramachandran Disallowed (%)**	0.00	0.00	0.00	0.00	0.00	0.00	0.00	0.00

Table S2. EC50 values in dCypher-Luminex binding assays

	EC50 relative (nM) *(Max MFI)*			
	Unmodified	H2A(E61A)	H2BK120ub	H2AK119ub
**GST-LANA**	192 *(66177)*	NB *(603)*	> 1300 *(14653)*	392 *(61989)*
**Chromatibody**	559 *(4748)*	NB *(182)*	419 *(979)*	544 *(5335)*
**1E9**	11 *(4546)*	NB *(126)*	NB *(152)*	10 *(5029)*
**1G1**	> 2000 *(584)*	NB *(37)*	NB *(85)*	> 2000 *(782)*
**1B2**	> 2000 *(978)*	NB *(27)*	NB *(55)*	> 2000 *(1332)*

EC50 relative calculated as above (4P Logistic; n=2; Graphpad Prism)
Max MFI is average MFI at maximum query concentration (n=2)
NB: no binding observed
